# Effects of Dietary Yeast β-1,3/1,6-D-Glucan on Immunomodulation in RAW 264.7 Cells and Methotrexate-Treated Rat Models

**DOI:** 10.3390/ijms252011020

**Published:** 2024-10-14

**Authors:** Joohee Son, Yeseul Hwang, Eun-Mi Hong, Marion Schulenberg, Hyungyung Chai, Hee-Geun Jo, Donghun Lee

**Affiliations:** 1 Department of Herbal Pharmacology, College of Korean Medicine, Gachon University, 1342 Seongnamdae-ro, Sujeong-gu, Seongnam-si 13120, Republic of Korea; 2 Department of Nutraceutical Ingredients Research, FINE BS Co., Ltd., 76 Yeonmujang-gil, Seongdong-gu, Seoul 04784, Republic of Korea; 3 Department of Product Management Nutraceuticals & Biotechnology, Leiber GmbH, Franz-Leiber-Straße 1, 49565 Bramsche, Germany; 4 Research Institute, Medicro Co., Ltd., Anyang 14067, Republic of Korea; 5 Naturalis Inc., 6, Daewangpangyo-ro, Bundang-gu, Seongnam-si 13549, Republic of Korea

**Keywords:** β-1,3/1,6-D-glucan, insoluble yeast β-glucan, immunomodulation, brewer’s spent yeast, immunoceuticals, nutraceuticals

## Abstract

A new subclass of nutraceuticals, called immunoceuticals, is dedicated to immunological regulation. Although yeast-derived β-1,3/1,6-D-glucan shows promise as an immunoceutical candidate, further studies are needed to define its precise immune-enhancing processes and to standardize its use. Following methotrexate (MTX)-induced immunosuppression in rats, we evaluated the immunomodulatory efficacy of a highly pure and standardized β-1,3/1,6-D-glucan sample (YBG) in RAW 264.7 macrophages. In in vitro and in vivo models, YBG demonstrated remarkable immunomodulatory effects, such as repair of immune organ damage, elevation of blood cytokine levels, and enhanced phagocytosis and nitric oxide production in RAW 264.7 cells. These results are consistent with the established immunostimulatory properties of β-glucan. It is noteworthy that this research indicates the potential of YBG as an immunomodulatory nutraceutical, as it is among the first to demonstrate immunological augmentation in an immunosuppression setting produced by MTX. Based on these observations, further investigation of YBG is warranted, particularly given its potential to emerge as a combination immunoceutical to mitigate immunosuppression and reduce the risk of infection in rheumatoid arthritis (RA) patients receiving long-term MTX therapy.

## 1. Introduction

In recent decades, a new trend in healthcare has emerged, emphasizing optimal health through diet and nutrition. The term “nutraceuticals”, a blend of “nutrition” and “pharmaceuticals”, has gained prominence as a research topic [1]. While there is no universally accepted definition, it is generally understood to refer to food-derived substances that provide health benefits beyond essential nutrition, such as physiological advantages or pharmacological effects on chronic conditions [2]. Recently, the subcategory of “immunoceuticals” has been introduced for nutraceuticals with immunomodulatory activity, particularly relevant in managing complex pathologies like cancer, infectious diseases, and autoimmune disorders, which often require long-term management [3]. This perspective is increasingly pertinent as nutraceuticals are recognized as potential preventive strategies against the recent COVID-19 pandemic and as adjuncts to immune checkpoint inhibitors in combating the rising incidence of various cancers [4,5]. Immunoceuticals include micronutrients such as vitamin D3, omega-3 fatty acids, zinc, and selenium, as well as various naturally occurring immunomodulatory compounds [3]. These have been recognized as alternative options to complement pharmaceuticals in mitigating and preventing immune-related pathological conditions, especially as the nutraceuticals market expands. However, developing immunoceuticals within a functional framework focused on “immunity enhancement” remains a challenge. There is still no consensus on specific targets and mechanisms of action, which complicates the establishment of clear guidelines for their use.

As mentioned above, significant strides have been made in immunotherapy, particularly in cancer treatment, leading to the development of various synthetic immunomodulators [6]. However, these synthetic agents are often criticized for their high costs and uncertain efficacy [7]. Consequently, natural product-derived immunomodulators have gained attention as a promising research area. Numerous phytoconstituents have shown significant immunomodulatory effects in both animal and human studies [8]. Polysaccharides, in particular, have attracted interest due to their broad pharmacological activities and potent immunomodulatory properties, comparable to those of high molecular weight compounds and proteins [9]. For example, a recent study highlighted the effects of *Astragalus mongholicus* Bunge, a medicinal plant commonly used in the treatment of cancer and autoimmune diseases [10]. The study showed that polysaccharides from this plant enhance the activity of macrophages, natural killer cells, dendritic cells, lymphocytes, and microglia through various cytokine and chemokine expression mechanisms. Among these polysaccharides, β-glucan, a homopolysaccharide composed of D-glucose, stands out as the most extensively studied for its immunomodulatory properties. It has recently emerged as one of the most promising candidates for immunoceuticals [11].

β-glucan is a component of the cell walls of plants, primarily wheat, as well as microorganisms such as yeasts and molds, providing structural organization and defense against pathogens and external factors [12,13,14]. All β-glucans share a β-1,3-glucan backbone, with mixed-linked β-glucans (1,3 and 1,4 linkages) present in plants, while microbial-derived β-glucans contain 1,3-glycosidic bonds and a smaller number of 1,6-glycosidic bonds [15]. Notably, only β-glucans from fungi or yeast with a high molecular weight and a high degree of branching exhibit immunomodulatory properties [16]. This is probably because cereal β-glucans containing only 1,3 or 1,4 glycosidic linkages are poorly absorbed and digested in the human small intestine [17]. In contrast, fungal or bacterial glucans with 1,6 branches can activate specific receptors, including Dectin-1, complement receptor 3 (CR3), and toll-like receptor 2 (TLR2). Recent studies have reported that yeast-derived β-glucan exhibits potent anticancer activity against hepatocellular carcinoma by inhibiting autophagic degradation [18]. Additionally, human clinical trials have shown that oral consumption of fungi-derived β-glucan significantly enhances immune responses by increasing the levels of immune cells such as CD3+, CD4+, and CD8+ T-lymphocytes, and improving CD4/CD8 ratio [19]. However, despite their promising potential, β-glucans exhibit wide structural variability, which has hindered their functional development [13]. Therefore, there is an urgent need to generate scientific evidence for immune-related biomarkers based on structurally standardized materials to advance the development of fungal-derived β-glucan immunoceuticals.

In line with the above, this study aimed to provide further reliable preclinical evidence of immune-related benefits of β-1,3/1,6-D-glucan. The sample used was the standardized β-1,3/1,6-D-glucan, extracted with high purity from spent brewer’s yeast (hereafter abbreviated YBG). Among the numerous β-glucans, YBG is specifically expected to possess potential in enhancing immune function due to its structural properties, which are essential for activating specific pattern recognition receptors (PRRs) involved in immune responses. YBG consists of a β-1,3-D-glucan oligosaccharides backbone, with a 1,6-β-linked side chain branch, a structural feature necessary for activating Dectin-1 receptors [20]. Furthermore, YBG is a high molecular weight particulate β-glucan, with a triple helix conformation and a branching ratio between 0.2 and 0.33 (1:5 to 1:3 branching), which is known to exhibit the most potent immunomodulatory properties [21]. We evaluated YBG using a comprehensive in vitro model to assess immune function and an in vivo model treated with methotrexate (MTX), the gold standard immunosuppressant [22]. The results of these investigations are reported below.

## 2. Results

### 2.1. Effects of YBG on Body Weights and Organ Index of Immunosuppressed Rats

**Figure 1:**
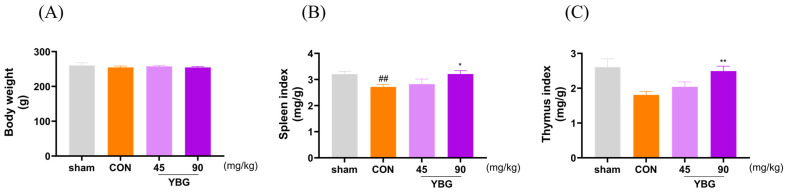
Effects of the YBG on body weight and organ indices in MTX-induced immunosuppressed rats. The SD rats were allocated into four groups, each consisting of eight animals. DW was utilized as a vehicle, and rats in YBG groups were given oral YBG (45, 90 mg/kg/day) once daily for 14 days. Subsequently, MTX (2 mg/kg/day, po) was administered for three consecutive days to induce immunosuppression. (**A**) The body weight of SD rats, (**B**) spleen index, and (**C**) thymus index. All results are shown as the mean ± SEM. ## *p* < 0.01 in comparison to the sham group; * *p* < 0.05 and ** *p* < 0.01 in comparison to the CON group by one-way ANOVA and Dunnett’s post hoc test. CON: control; YBG: yeast β-glucan; MTX: methotrexate.

The body weights were measured daily to determine the effect of MTX-induced immunosuppression and YBG treatment (45, 90 mg/kg) on rats (Figure 1). The indices of immune-related organs were measured to investigate the protective effect of YBG on immunosuppressed rats. Immunosuppression did not lead to any notable alterations in body weight; the organ indices showed a significant decline in comparison with the results for the sham group. YBG administration mitigated the damaging effects of MTX on the spleen (*p <* 0.05) and thymus (*p <* 0.01) compared to those of the control group. The YBG 90 mg/kg group exhibited a protective effect that resulted in levels comparable to those of the sham group.

### 2.2. Effects of YBG on Hematological Parameters of Immunosuppressed Rats

**Figure 2:**
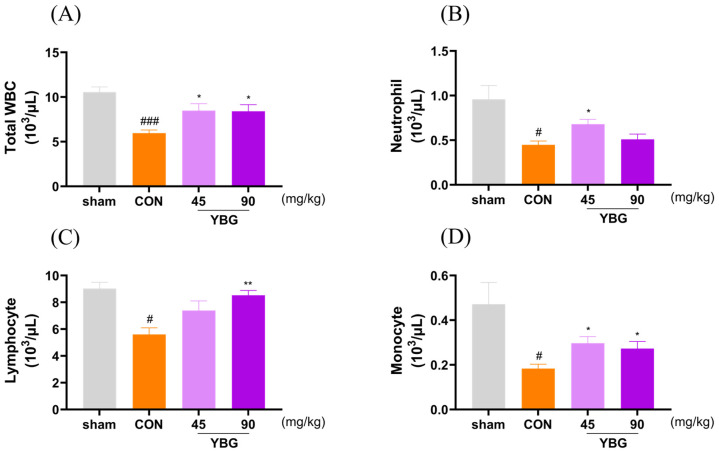
Effects of YBG on blood leukocyte number in MTX-induced immunosuppressed rats. SD rats were treated with distilled water or oral YBG (45, 90 mg/kg/day) for 14 days. Subsequently, MTX (2 mg/kg/day, po) was given for 3 consecutive days to induce immunosuppression. (**A**) Total white blood cells, (**B**) neutrophils, (**C**) lymphocytes, and (**D**) monocytes. All results are shown as mean ± SEM. # *p* < 0.05 and ### *p* < 0.001 in comparison to the sham group; * *p* < 0.05 and ** *p* < 0.01 in comparison to the CON group by one-way ANOVA and Dunnett’s post hoc test. CON: control; YBG: yeast β-glucan; MTX: methotrexate.

To evaluate the effect of YBG on blood circulating white blood cell contents, we conducted a hematological analysis (Figure 2). MTX treatment significantly decreased total white blood cell numbers (WBC), as well as the number of lymphocytes, neutrophils, and monocytes compared to those of the sham group. YBG administration restored the total white blood cell numbers in both the YBG 45 and YBG 90 groups when compared with the results for the control group (*p* < 0.05). Notably, YBG treatment also elevated the counts of leukocyte subtypes, including neutrophils (*p* < 0.05), lymphocytes (*p* < 0.01), and monocytes (*p* < 0.05), as compared with those of the control group.

### 2.3. Effects of YBG on Splenocyte Proliferation of Immunosuppressed Rats

**Figure 3:**
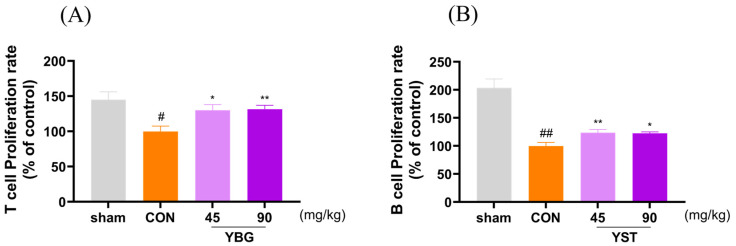
Effects of YBG on splenocyte proliferation induced by Con-A and LPS in MTX-induced immunosuppressed rats. SD rats were given saline or oral YBG (45, 90 mg/kg/day) for 14 days. Subsequently, MTX (2 mg/kg/day, po) was administered orally for 3 consecutive days to induce immunosuppression. (**A**) Con-A induced T lymphocyte proliferation; (**B**) LPS induced B lymphocyte proliferation. All results are shown as mean ± SEM. # *p* < 0.05 and ## *p* < 0.01 in comparison to the sham group, * *p* < 0.05 and ** *p* < 0.01 in comparison to the CON group by one-way ANOVA and Dunnett’s post hoc test. CON: control; YBG: yeast β-glucan; MTX: methotrexate;.

To assess the effect of YBG on lymphocyte function, the proliferation rates of splenocytes were examined (Figure 3). Con-A was utilized to induce T lymphocyte proliferation, and LPS was administered as a stimulant for B lymphocyte. A significant decline in the proliferative activity of both the T and B lymphocytes was observed in the control group compared with that of the sham group. The oral administration of YBG significantly restored the diminished proliferative activity in both the T and B lymphocytes compared to that of the control group. Notably, YBG treatment provided greater protective effects to the T lymphocytes than the B lymphocytes, restoring their proliferation to levels comparable to those of the sham group.

### 2.4. Effects of YBG on NK Cell Activity of Immunosuppressed Rats

**Figure 4:**
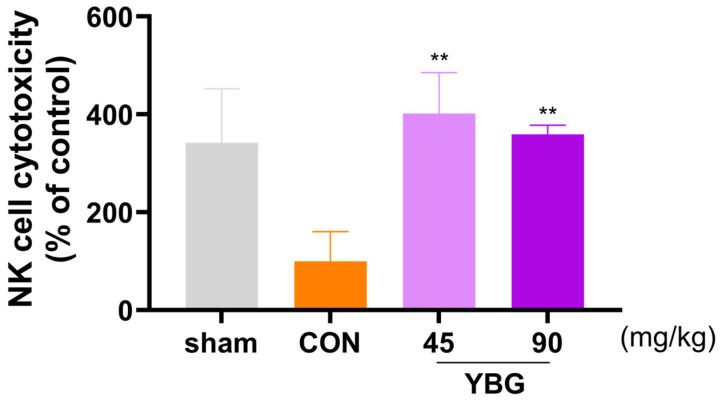
Effects of YBG on NK cell activity in MTX-induced immunosuppressed rats. SD rats were administered distilled water or oral YBG (45, 90 mg/kg/day) for 14 days. Subsequently, MTX (2 mg/kg/day, po) was given for 3 consecutive days to induce immunosuppression. NK-sensitive fibroblast, YAC-1 cells were utilized as target cells. All results are shown as mean ± SEM. ** *p* < 0.01 in comparison to the CON group by one-way ANOVA and Dunnett’s post hoc test. CON: control; YBG: yeast β-glucan; MTX: methotrexate.

Natural killer cell activity was evaluated to determine the effects of YBG on cell mediated non-specific innate immunity (Figure 4). To assess the NK cell activity, the cytolytic activity of splenocytes against the YAC-1 cells was evaluated. Compared to the sham group, the immunosuppressed control group exhibited decreased NK cell activity. Conversely, the NK cell activity significantly rose in the YBG administration groups compared to that of the control group (*p* < 0.01).

### 2.5. Effects of YBG on Cytokine Productions of Immunosuppressed Rats

**Figure 5:**
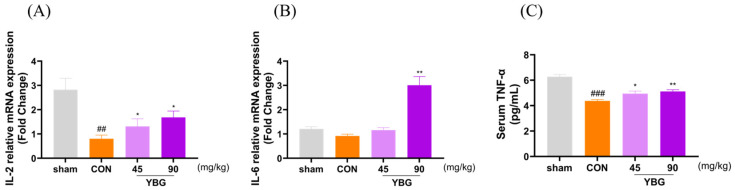
The effect of YBG on cytokine release in MTX-induced immunosuppressed rats. SD rats were given distilled water or oral YBG (45, 90 mg/kg/day) for 14 days. Subsequently, MTX (2 mg/kg/day) was administrated orally for 3 days to induce immunosuppression. (**A**) mRNA expression of IL-2 and (**B**) IL-6 in the spleen; (**C**) serum TNF-α level. qRT-PCR was utilized to examine the mRNA expression levels of IL-2 and IL-6, while serum TNF-α levels were measured using ELISA. The data are shown as mean ± SEM. ## *p* < 0.01 and ### *p* < 0.001 in comparison to the sham group; * *p* < 0.05 and ** *p* < 0.01 in comparison to the CON group by one-way ANOVA and Dunnett’s test; CON: control; YBG: yeast β-glucan; MTX: methotrexate; IL: interleukin; TNF: tumor necrosis factor.

To evaluate the effects of YBG on pro-inflammatory cytokine production, the mRNA expression levels of IL-2 and IL-6 in the spleen and serum TNF-α levels were measured. (Figure 5). Immunosuppression led to a significant reduction in serum TNF-α levels and mRNA expression of IL-2 and IL-6 in the spleen compared to that in the sham group. YBG administration, in contrast, reversed the reduction in serum TNF-α levels and spleen mRNA expression of IL-6 and IL-2, showing a dose-dependent effect compared to that in the control group. 

### 2.6. Effects of YBG on Serum Immunoglobulin Levels of Immunosuppressed Rats

**Figure 6:**
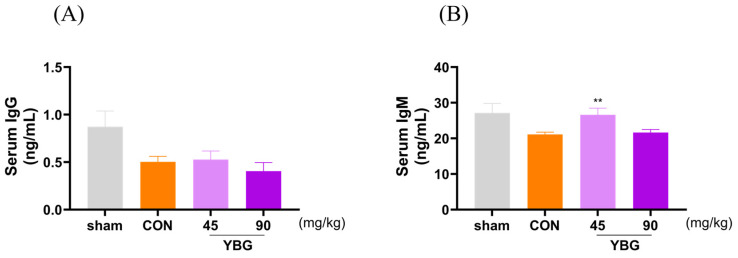
The effect of YBG on immunoglobulin production in MTX-induced immunosuppressed rats. SD rats were given distilled water or oral YBG (45, 90 mg/kg/day) for 14 days. Subsequently, MTX (2 mg/kg/day) was orally administered for 3 days to induce immunosuppression. (**A**) IgG and (**B**) IgM levels in the serum were estimated using ELISA. All results are shown as mean ± SEM. ** *p* < 0.01 in comparison to the CON group by one-way ANOVA and Dunnett’s test. CON: control; YBG: yeast β-glucan; Ig: immunoglobulin.

To examine the effect of YBG on humoral immune responses, serum immunoglobulin levels were measured (Figure 6). IgG is the predominant immunoglobulin, and IgM levels are considered biomarkers for the initial immune response against infection. Therefore, the levels of these two immunoglobulins were measured. The immunosuppression led to reduced serum IgG and IgM levels relative to the sham group. While there were no differences in the serum IgG levels between the YBG groups and the control group, the YBG 45 group showed a significant rise in serum IgM levels when compared with those of the control group (*p <* 0.01).

### 2.7. Effects of YBG on Cell Viability and Phagocytosis of RAW 264.7

**Figure 7:**
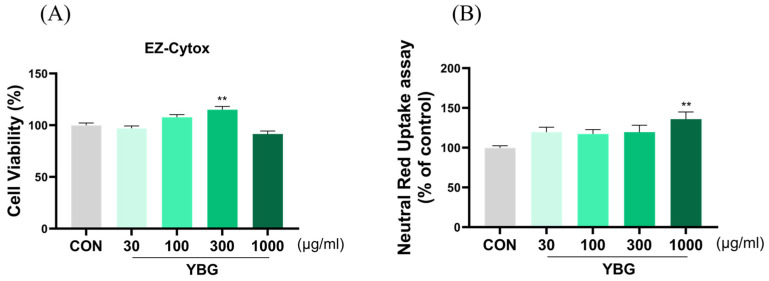
Effects of the YBG on cell viability and phagocytic activity in RAW 264.7. (**A**) cell viability and (**B**) phagocytosis. The phagocytic activity was examined using a neutral red uptake assay. All results are shown as mean ± SEM. ** *p* < 0.01 in comparison to the CON group by one-way ANOVA and Dunnett’s test. CON: control; YBG: yeast β-glucan.

To determine the toxicity of YBG, RAW 264.7 cells were exposed to varying doses of YBG (30–1000 µg/mL). YBG exhibited no toxic effects and interestingly, led to a significant increase in cell viability at 300 μg/mL (*p <* 0.01) (Figure 7). To evaluate the effect of YBG on the RAW 264.7 cells phagocytosis, a neutral red uptake activity assay was conducted. YBG improved phagocytic activity starting at a dose of 30 μg/mL; a significant increase was observed at a dose of 1000 μg/mL (*p <* 0.01).

### 2.8. Effects of YBG on Nitric Oxide and Cytokine Productions in RAW 264.7

**Figure 8:**
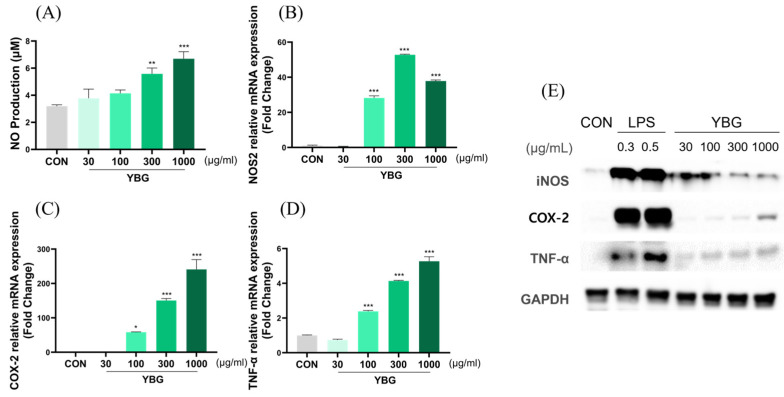
Effects of the YBG on nitric-oxide production and proinflammatory factors of RAW 264.7. (**A**) NO production and mRNA expressions of (**B**) NOS2, (**C**) COX-2, and (**D**) TNF-α; (**E**) protein secretion of iNOS, COX-2, and TNF- α from RAW 264.7. The mRNA expression levels were examined using qRT-PCR, while protein productions were estimated using a Western blot assay. All results are shown as mean ± SEM. * *p* < 0.05, ** *p* < 0.01, and *** *p* < 0.001 in comparison to the CON group by one-way ANOVA and Dunnett’s test, CON: control; YBG: yeast β-glucan; NOS2: nitric oxide synthase 2; COX-2: cyclooxygenase 2; TNF: tumor necrosis factor.

To evaluate the effects of YBG on proinflammatory factors in RAW 264.7 cells, NO production was estimated. The protein production and mRNA expression levels of NOS2, TNF-α, and COX-2 were examined to determine the factors that YBG modulates (Figure 8). YBG treatment led to a significant and dose-dependent enhancement in the production of NO, beginning at a dose of 300 μg/mL. Aligning with the rise in NO secretion, the mRNA level of NOS2, TNF-α, and COX-2 significantly increased in cells administered a dose of 100, 300, and 1000 μg/mL of YBG.

## 3. Discussion

As demonstrated by the results, YBG increased the spleen and thymus indices in the MTX-induced immunosuppression rat model and significantly enhanced splenocyte proliferation. Additionally, YBG improved NK cell activity and dose-dependently increased the production of immunological cytokines such as IL-2, IL-6, and TNF-α, while significantly raising the total WBC count, lymphocytes, and monocytes. In vitro studies consistently replicated these immunomodulatory effects. YBG treatment enhanced phagocytosis and NO production in RAW 264.7 cells and dose-dependently upregulated the expression of pro-inflammatory factors, including NOS2, COX-2, and TNF-α. The following is a discussion of the implications of these results.

The observation that YBG increased the spleen and thymus index in rats atrophied by MTX administration is noteworthy. This finding highlights YBG’s immunomodulatory potential and suggests its broader applicability as an immunoceutical. By reversing MTX-induced organ atrophy, YBG may enhance immune function, positioning it as a promising candidate for therapies aimed at modulating immune responses in conditions of immunosuppression. MTX attenuates the immune response primarily by inhibiting dihydrofolate reductase, which prevents the conversion of dihydrobiopterin to tetrahydrobiopterin. MTX especially interferes with the DNA synthesis of cells in the S phase, thereby suppressing the proliferation of fast-dividing cells such as immune cells and cancer cells [22]. This reaction also induces the uncoupling of NOS, hinders the production of NO, and enhances the apoptotic sensitivity of T cells, thereby modulating the immune response [23]. Additionally, MTX contributes to immunosuppression by increasing adenosine release, activating the adenosine receptor, and by inhibiting the activity of nuclear factor-κB, a key regulator of immune reactions. The immunosuppressive effects of MTX in rheumatoid arthritis (RA) patients are well-documented, often leading to increased susceptibility to infections or the exacerbation of infectious diseases [24]. Reports indicate that MTX-treated RA patients face a higher incidence of various bacterial and opportunistic infections, including cryptococcosis, cytomegalovirus pneumonia, herpes simplex hepatitis, and Pneumocystis carinii pneumonia [24]. These complications not only hinder the achievement of RA treatment goals but also pose significant health risks [25,26]. The immunosuppressive effects of MTX parallel those of secondary immunodeficiency, as it transiently impairs immune cells and tissues, disrupts both cellular and antibody responses, and inhibits proinflammatory cytokine production [27]. However, the potential of nutraceuticals to mitigate these adverse effects is promising. For example, folic acid supplementation has been shown to reduce MTX-related side effects [28]. In light of these findings, the significant immunomodulatory effects of YBG observed in this study, particularly its impact on splenocytes, including T and B lymphocytes, suggest that YBG could be a valuable immunoceutical. Further in-depth studies could explore YBG’s potential as a combination immunoceutical for alleviating infections and supporting patients with secondary immunodeficiency caused by various conditions, including the use of immunosuppressive medications, malnutrition, metabolic disorders, and malignancies.

The proliferative effects of YBG on T and B lymphocytes observed in this study were consistent with the existing literature on β-glucan’s role in immune modulation. Previous research has demonstrated that β-glucan activates dendritic cells (DCs), promoting the priming and differentiation of Th1 and cytotoxic T lymphocytes. This process enhances adaptive immune responses by increasing the production of innate cytokines and inducing autophagosome formation [29]. Furthermore, β-glucan has been shown to activate circulating B lymphocytes via the Dectin-1 receptor and regulate IL-1β secretion through the NLRP3 inflammasome [30]. These findings suggest that the β-glucan component of YBG contributes significantly to its immunomodulatory properties, specifically by enhancing the activity of the circulating blood cells and NK cells. This supports the view that YBG offers beneficial functions in regard to immune regulation, reinforcing its potential as an adjunctive therapy to mitigate immunosuppressive side effects in treatments like those involving MTX. The observed effects of YBG on NK cells in this study closely resemble the action of β-1,3/1,6-D-glucan in modulating macrophage phenotype [31]. When administered orally, β-glucan is processed by gastrointestinal macrophages and broken down into smaller fragments that are subsequently recognized by the Dectin-1 receptor on the macrophages [32]. Dectin-1 is a well-known pattern recognition receptor for β-glucan, playing a critical role in various immune functions, including activating DC, enhancing macrophage phagocytosis, and differentiating T lymphocytes. Recent research has expanded our understanding of Dectin-1, revealing its direct involvement in regulating NK cell activity in response to β-glucan [31]. The potential immunomodulatory properties of YBG are supported by widely reported studies suggesting that the antitumor effects of β-glucan may be related to NK cells.

At the same time, this process involves phenotypic reprogramming of tumor-associated macrophages by β-glucan, which can enhance NK cell-mediated anti-tumor activity [33,34]. The findings in this manuscript suggest that YBG’s effects on NK cells and macrophages could contribute to its broader immunotherapeutic potential, particularly in the context of cancer and chronic inflammatory conditions.

To cross-validate the immunomodulatory effects of YBG, we utilized an in vitro model involving RAW 264.7 macrophages, a well-established cell line for evaluating immune function, as it can assess both immune-stimulatory and anti-inflammatory activities [35]. Our study demonstrated that YBG enhances the phagocytic ability of RAW 264.7 cells and increases NO production, which are key indicators of macrophage activation and immune function. Phagocytosis is a crucial defense mechanism in which macrophages ingest and eliminate antigens, protecting the body from infections. NO, produced through inducible nitric oxide synthase, is a potent effector molecule used by macrophages to destroy foreign microorganisms [36]. The observed increases in macrophage phagocytosis and NO production suggest that YBG effectively stimulates the innate immune response, consistent with the results of previous studies using similar materials [11,13,19,31]. This macrophage activation involves the upregulation of the nuclear factor-κB(NF-κB) signaling pathway, a critical regulator of the immune response. NF-κB activation induces the secretion of various cytokines and immune mediators, including NOS2, TNF-α, and COX-2 [35,36]. In support of YBG’s role in enhancing immune function, these responses were clearly observed in our in vitro experiments. Furthermore, the immune factor activity induced by YBG in RAW 264.7 cells is consistent with the increased levels of IL-2, IL-6, and TNF-α observed in the spleen and serum of immunosuppressed rats treated with YBG. These cytokines play pivotal roles in innate and adaptive immunity, further corroborating YBG’s immunomodulatory potential. Natural products known for their immune-enhancing properties, such as probiotics, *Cordyceps militaris* polysaccharide, and *Astragalus* polysaccharide, have also been evaluated for their biological activities in RAW 264.7 cells. In comparison to the results of these studies, YBG demonstrated similar or greater activities in stimulating phagocytosis, NO production, and the mRNA expression of pro-inflammatory cytokines of RAW 264.7 [10,37,38]. Collectively, our data suggest that YBG has significant modulatory effects on both innate and adaptive immune responses, as demonstrated by both in vivo and in vitro models.

These findings provide a strong foundation for further exploration of YBG as an immunoceutical, with potential applications in enhancing immune function and managing immune-related conditions. Despite the positive observations for YBG, such as its ability to modulate multifaceted immunity, this study has several limitations that necessitate further research before the findings can be extrapolated to humans. First and foremost, additional studies are required to determine the optimal dosage of YBG to fully harness its potential as an immunotherapeutic agent for MTX-treated RA, as suggested by our results. Furthermore, future research should employ target-specific investigations, along with improved experimental models and methodologies to investigate whether YBG can effectively modulate the highly complex pathophysiological mechanism of immune dysregulation when used as a combination therapy. A follow-up study that addresses the limitations mentioned above is anticipated to demonstrate the efficacy of β-glucan as an adjunct therapy for patients undergoing MTX treatment. Additionally, our findings suggest that YBG may have the potential to enhance immunity in individuals exhibiting broader immunosuppressive conditions.

## 4. Materials and Methods

All experiments conducted in this study were performed in compliance with the ARRIVE 2.0 guidelines [39].

### 4.1. Preparation of β-Glucan

The standardized yeast β-1,3/1,6-D-glucan (YBG) is extracted from the cell wall of spent brewer’s yeast (*Saccharomyces cerevisiae*) [14]. The β-glucan preparation used in the study is Yestimun^®^, a commercially available, insoluble, and well-characterized β-glucan, and was provided by FINE-BS (Seoul, Republic of Korea), with Leiber GmbH (Bramsche, Germany) serving as the original manufacturer. The preparation process followed the protocol described in a previous report. Briefly, yeast underwent autolysis using its own enzyme and was then centrifuged to separate the insoluble elements from the cell wall. These components were treated with a weak alkali, avoiding the use of acid to maintain the (1 → 3) and (1 → 6) bonds, resulting in the collection of insoluble yeast β-1,3/1,6-D-glucan. The β-glucan preparation contained β-1,3/1,6-D-glucan, with a purity of at least 80%, as measured using the Enzymatic Yeast β-Glucan Assay Kit (Megazyme K-EBHLG, Bray, Ireland). YBG demonstrated an average relative linkage ratio of 22% from β-1,6 glucan. It also contained alpha-D-mannan < 2.0%, protein < 4.0%, and fat < 3.0%. The detailed composition is presented in Supplementary Materials.

### 4.2. Animal and Experimental Designs

Six-week-old male Sprague Dawley (SD) rats (150–170 g, *n* = 32) were obtained from DBL Co., Ltd. (DBL, Paju-si, Republic of Korea) and used to establish MTX-induced immunosuppressed models. The rats were submitted to a 7-day acclimation under normal laboratory conditions, kept on a 12 h day/night cycle at 22 ± 2 °C with a humidity of 45 ± 10%, and allowed to freely access water and food. The study followed the guidelines established by the Gachon University Center for Animal Care and Use for all procedures (GU1-2023-IA0082-00). The rats were randomly allocated into four groups: sham, control (CON), YBG 45, and YBG 90, with each group consisting of eight rats. Distilled water (DW) was utilized as a vehicle, and the rats in the YBG groups were orally administered YBG (45 and 90 mg/kg/day). The dosage of β-glucan conformed to the amounts used in previous studies, including both animal studies and clinical trials [14]. For the clinical trial, the dosage was adjusted according to the conversion rate from humans to rats [40]. All samples and the vehicle were administered orally for 14 days, and the control and YBG groups were orally administered MTX at 2 mg/kg for 3 days to induce immunosuppression. The day after the final administration of MTX, all rats were euthanized using CO₂, and blood, spleen, and thymus tissues were collected.

### 4.3. Measurement of Organ Index

The spleens and thymus tissues were removed under sterile conditions and weighed upon collection. The spleen and thymus indices are estimated as the organ weight divided by the body weight.

### 4.4. Hematological Analysis

Whole blood drawn from the abdominal vena cava was transferred into vials containing 0.1 M EDTA (BD Vacutainer, Franklin Lakes, NJ, USA). The samples were immediately used for the analysis. Erythrocytes (RBC), platelets (PLT), total leukocytes (WBC), and differential white blood cell counts—including neutrophils, lymphocytes, and monocytes—were assessed using an XN 1000 automated hematologic analyzer (Sysmex, Kobe, Japan).

### 4.5. Spleen Lymphocyte Proliferation Analysis

The spleens of the mice were collected and immediately placed in ice-cold PBS until the isolation. The spleens were gently ground in cold PBS and filtered using 70 μm mesh strainers (SPL Life Sciences, Po-cheon, Republic of Korea). Splenocytes were extracted from the cell suspension using RBC Lysis Buffer (Sigma-Aldrich, Burbank, CA, USA). The extracted splenocytes were grown in RPMI-1640 medium enriched with 10% fetal bovine serum and 1% P/S (Gibco BRL, Carlsbad, CA, USA). The splenocytes from each rat were then seeded into a 96-well plate (4.0 × 10^5^ cells/well). Con-A at a dose of 5 μg/mL was used as a T lymphocyte stimulant, while LPS at 10 μg/mL served as a B lymphocyte mitogen. The splenocytes were incubated in the presence of Con-A and LPS for 24 h at 37 °C, 5% CO_2_. To assess the proliferation rate, each well was treated with 25 μL of EZ-Cytox solution, then incubated for an additional 1 h. Absorbance measurements were conducted at 450 nm.

### 4.6. Natural Killer Cell Activity Measurement

YAC-1 (mouse fibroblast) was used as the target cell for NK cell activity measurement. YAC-1 cells were grown in RPMI-1640 medium enriched with 10% fetal bovine serum and 1% P/S (Gibco BRL, Carlsbad, CA, USA). The isolated splenocytes of each rat were utilized as effector cells. The YAC-1 cells (1 × 10^4^/well) were co-cultured with splenocyte (2.5 × 10^5^/well) at the ratio of 25:1 in U-bottom 96-well plates and incubated for 4 h. For measuring the spontaneous release of the effector cells, splenocyte (2.5 × 10^5^/well) in RPMI-1640 media was seeded into another U-bottom 96-well plate. For measuring the spontaneous release of the target cells, the YAC-1 (1 × 10^4^/well) and RPMI-1640 media were seeded. To determine the maximum release of the target cells, YAC-1 (1 × 10^4^/well) with cell lysate buffer was added. The supernatants were moved into a new 96-well plate and incubated with 60 μL of EZ-LDH reagent (DoGen-Bio, Seoul, Republic of Korea). Absorbance measurement was conducted at 490 nm. The NK cell activities were estimated using the following formula: Lysis (%) = [(OD of co-cultured group − OD of effector cells spontaneous releases − OD of target cells spontaneous releases)/(OD of Target cells maximum releases − OD of target cells spontaneous releases)] × 100. The data are presented as values (%) relative to the control.

### 4.7. Serum Cytokine and Immunoglobulin Analysis

Whole blood extracted from the abdominal vena cava was placed into plain sample tubes and left for 30 min to allow for clot formation. The serum was obtained by centrifuging the samples for 10 min at 4000 rpm and subsequently maintained at −80 °C until further use. Serum cytokine levels were measured using the TNF-α ELISA kit (KE20018, Proteintech, Rosemont, IL, USA). Serum immunoglobulin levels were assessed using the IgG ELISA kit (ab189578, Abcam, Danvers, MA, USA) and the IgM ELISA kit (ab215085, Abcam, Danvers, MA, USA). All experimental methods were performed while following the manufacturer’s protocols.

### 4.8. q-RT PCR Analysis

Total RNAs were obtained from RAW 264.7 cells and spleen tissues using TaKaRa Mini-BEST Universal RNA Extraction Kits (TaKaRa Bio Korea, Seoul, Republic of Korea). The cDNA synthesis was performed with Convert Mix (Bioneer, Dae-jeon, Republic of Korea). qRT-PCR was performed using Exicycler 96 (Bioneer, Dae-jeon, Republic of Korea). Accu-Power 2× Green-Star qPCR Master Mix (Bioneer, Dae-jeon, Republic of Korea) was used to amplify the cDNA. All processes were carried out following the protocols offered by the manufacturer. The primer sequences utilized in the investigation are indicated in Table 1 and Table 2. 

### 4.9. Cell Culture and Cell Viability Assay

RAW 264.7 macrophages obtained from the Korean Cell Line Bank (Seoul, Republic of Korea) were grown in DMEM medium enriched with 10% FBS and 1% P/S (Gibco BRL, Carlsbad, CA, USA). To estimate the cytotoxicity of YBG, RAW 264.7 cells (5.0 × 10^5^ cells/well) were placed in a 96-well plate and cultured with varying doses of YBG (30–1000 µg/mL) for 24 h at 37 °C with 5% CO_2_. The measurements of cell viability were conducted using Ez-Cytox solution (DoGen-Bio, Seoul, Republic of Korea). Absorbance measurement was conducted at 450 nm. The experiment was conducted three times, and the outcomes were shown as values (%) relative to the control.

### 4.10. Phagocytosis Assay

RAW 264.7 cells (5.0 × 10^5^ cells/well) were cultured with varying doses of YBG (30–1000 µg/mL) in a 96-well plate. Following a 24 h incubation, the culture media was substituted with DMEM 100 μL containing 10% neutral red reagent (Sigma, Burbank, CA, USA) and then incubated for 4 h. The cells were then washed with 0.1 M PBS to eliminate any excess dye solution. Finally, 150 μL of cell lysis solution (acetic acid and ethanol in a 1:1 volume ratio) was added. The absorbance was recorded at 540 nm.

### 4.11. NO Production Assay

RAW 264.7 cells (5.0 × 10^5^ cells/well) were cultured with varying doses of YBG (30–1000 µg/mL) in a 96-well plate for 24 h. To estimate the NO production, Greiss reagent was combined with the supernatant. The absorbance measurement was conducted at 540 nm.

### 4.12. Statistical Analysis

One-way ANOVA was conducted to investigate the effect of the samples on the dependent variable. The analysis of the statistics was performed using Graph-Pad Prism (ver. 10.0, Graph-Pad Software, San Diego, CA, USA). To further elucidate the specificity of the effect of each dose on the sample, Dunnet’s test was conducted as an additional post hoc analysis. This hypothesis-testing method was employed to ascertain whether at least one sample demonstrated superior efficacy in comparison to that of the control group. It was deemed that this method was more suitable for this study than the Tukey–Kramer method, as it can offset the reduction in statistical power resulting from multiple tests. All results were shown as mean ± standard error of the mean (SEM), and a *p*-value below 0.05, 0.01, or 0.001 was regarded as statistically significant.

## 5. Conclusions

In conclusion, the results of this study demonstrate that YBG, a standardized and well-purified yeast β-1,3/1,6-D-glucan, effectively improves immune function in both MTX-induced immunosuppressed rat models and RAW 264.7 cells. YBG exhibited consistent and relevant modulatory effects on innate and adaptive immune responses in both in vivo and in vitro studies. These findings suggest that YBG holds promising potential as an immunoceutical to counteract various immunosuppressive conditions. Further studies examining the infection risk in RA patients undergoing MTX treatment will be valuable for refining the therapeutic potential of YBG in clinical settings. Additionally, its effects on a broader range of immunosuppressive conditions should be validated through comprehensive experiments.

## Figures and Tables

**Table 1:** Primer sequences for spleen tissues of MTX-induced immunosuppressed rats.

Gene	Primer	Seqeunce
GAPDH	F	CTTGTGACAAAGTGGACATTGTT
	R	TGACCAGCTTCCCATTCTC
IL-2	F	CTCCCCATGATGCTCACGTT
	R	TCCAGCGTCTTCCAAGTGAA
IL-6	F	TCCGCAAGAGACTTCCAGC
	R	GCCGAGTAGACCTCATAGTGACC

GAPDH: glyceraldehyde-3-phosphate dehydrogenase; IL: interleukin.

**Table 2:** Primer sequences for RAW 264.7 cells.

Gene	Primer	Seqeunce
GAPDH	F	CTTGTGACAAAGTGGACATTGTT
	R	TGACCAGCTTCCCATTCTC
TNF-α	F	GAGAAGTTCCCAAATGGCCT
	R	AGCCACTCCAGCTGCTCCT
COX-2	F	ATCCATGTCAAAACCGTGGG
	R	TTGGGGTGGGCTTCAGCAG
NOS2	F	ACCAAGATGGCCTGGAGGAA
	R	CCGACCTGATGTTGCCATTG

GAPDH: glyceraldehyde-3-phosphate dehydrogenase; TNF: tumor necrosis factor; NOS2: nitric oxide synthase2; COX-2: cyclooxygenase 2.

## Data Availability

All data from this study are included in the main body of the article.

## Supplementary Materials

The following supporting information can be downloaded at: https://www.mdpi.com/article/10.3390/ijms252011020/s1.

## References

[1] Fernandes F.A., Carocho M., Prieto M.A., Barros L., Ferreira I.C.F.R., Heleno S.A. (2024). Nutraceuticals and Dietary Supplements: Balancing out the Pros and Cons. Food Funct..

[2] Chopra A.S., Lordan R., Horbańczuk O.K., Atanasov A.G., Chopra I., Horbańczuk J.O., Jóźwik A., Huang L., Pirgozliev V., Banach M. (2022). The Current Use and Evolving Landscape of Nutraceuticals. Pharmacol. Res..

[3] Tieu S., Charchoglyan A., Wagter-Lesperance L., Karimi K., Bridle B.W., Karrow N.A., Mallard B.A. (2022). Immunoceuticals: Harnessing Their Immunomodulatory Potential to Promote Health and Wellness. Nutrients.

[4] Zhong Z., Vong C.T., Chen F., Tan H., Zhang C., Wang N., Cui L., Wang Y., Feng Y. (2022). Immunomodulatory Potential of Natural Products from Herbal Medicines as Immune Checkpoints Inhibitors: Helping to Fight against Cancer via Multiple Targets. Med. Res. Rev..

[5] Zaman R., Ravichandran V., Tan C.K. (2024). Role of Dietary Supplements in the Continuous Battle against COVID-19. Phytother. Res..

[6] Zhang Y., Zhang Z. (2020). The History and Advances in Cancer Immunotherapy: Understanding the Characteristics of Tumor-Infiltrating Immune Cells and Their Therapeutic Implications. Cell. Mol. Immunol..

[7] Couchoud C., Fagnoni P., Aubin F., Westeel V., Maurina T., Thiery-Vuillemin A., Gerard C., Kroemer M., Borg C., Limat S. (2020). Economic Evaluations of Cancer Immunotherapy: A Systematic Review and Quality Evaluation. Cancer Immunol. Immunother..

[8] Ali S.A., Singh G., Datusalia A.K. (2021). Potential Therapeutic Applications of Phytoconstituents as Immunomodulators: Pre-Clinical and Clinical Evidences. Phytother. Res..

[9] Rong X., Shen C., Shu Q. (2024). Interplay between Traditional Chinese Medicine Polysaccharides and Gut Microbiota: The Elusive “Polysaccharides-Bond-Bacteria-Enzyme” Equation. Phytother. Res..

[10] Li C.-X., Liu Y., Zhang Y.-Z., Li J.-C., Lai J. (2022). Astragalus Polysaccharide: A Review of Its Immunomodulatory Effect. Arch. Pharm. Res..

[11] Zhong X., Wang G., Li F., Fang S., Zhou S., Ishiwata A., Tonevitsky A.G., Shkurnikov M., Cai H., Ding F. (2023). Immunomodulatory Effect and Biological Significance of β-Glucans. Pharmaceutics.

[12] Mizuno M., Minato K.-I. (2024). Anti-Inflammatory and Immunomodulatory Properties of Polysaccharides in Mushrooms. Curr. Opin. Biotechnol..

[13] De Marco Castro E., Calder P.C., Roche H.M. (2021). β-1,3/1,6-Glucans and Immunity: State of the Art and Future Directions. Mol. Nutr. Food Res..

[14] Stier H., Ebbeskotte V., Gruenwald J. (2014). Immune-Modulatory Effects of Dietary Yeast Beta-1,3/1,6-D-Glucan. Nutr. J..

[15] Fang X.-H., Zou M.-Y., Chen F.-Q., Ni H., Nie S.-P., Yin J.-Y. (2021). An Overview on Interactions between Natural Product-Derived β-Glucan and Small-Molecule Compounds. Carbohydr. Polym..

[16] van Steenwijk H.P., Bast A., de Boer A. (2021). Immunomodulating Effects of Fungal Beta-Glucans: From Traditional Use to Medicine. Nutrients.

[17] Wani S.M., Gani A., Mir S.A., Masoodi F.A., Khanday F.A. (2021). β-Glucan: A Dual Regulator of Apoptosis and Cell Proliferation. Int. J. Biol. Macromol..

[18] Wang N., Liu H., Liu G., Li M., He X., Yin C., Tu Q., Shen X., Bai W., Wang Q. (2020). Yeast β-D-Glucan Exerts Antitumour Activity in Liver Cancer through Impairing Autophagy and Lysosomal Function, Promoting Reactive Oxygen Species Production and Apoptosis. Redox Biol..

[19] Chen S.-N., Nan F.-H., Liu M.-W., Yang M.-F., Chang Y.-C., Chen S. (2023). Evaluation of Immune Modulation by β-1,3; 1,6 D-Glucan Derived from *Ganoderma lucidum* in Healthy Adult Volunteers, A Randomized Controlled Trial. Foods.

[20] Adams E.L., Rice P.J., Graves B., Ensley H.E., Yu H., Brown G.D., Gordon S., Monteiro M.A., Papp-Szabo E., Lowman D.W. (2008). Differential High-Affinity Interaction of Dectin-1 with Natural or Synthetic Glucans Is Dependent upon Primary Structure and Is Influenced by Polymer Chain Length and Side-Chain Branching. J. Pharmacol. Exp. Ther..

[21] Han B., Baruah K., Cox E., Vanrompay D., Bossier P. (2020). Structure-Functional Activity Relationship of β-Glucans From the Perspective of Immunomodulation: A Mini-Review. Front. Immunol..

[22] Cronstein B.N., Aune T.M. (2020). Methotrexate and Its Mechanisms of Action in Inflammatory Arthritis. Nat. Rev. Rheumatol..

[23] Janaszak-Jasiecka A., Płoska A., Wierońska J.M., Dobrucki L.W., Kalinowski L. (2023). Endothelial Dysfunction Due to eNOS Uncoupling: Molecular Mechanisms as Potential Therapeutic Targets. Cell. Mol. Biol. Lett..

[24] Wang W., Zhou H., Liu L. (2018). Side Effects of Methotrexate Therapy for Rheumatoid Arthritis: A Systematic Review. Eur. J. Med. Chem..

[25] Ibrahim A., Ahmed M., Conway R., Carey J.J. (2018). Risk of Infection with Methotrexate Therapy in Inflammatory Diseases: A Systematic Review and Meta-Analysis. J. Clin. Med..

[26] Tidblad L., Westerlind H., Delcoigne B., Askling J., Saevarsdottir S. (2023). Comorbidities and Chance of Remission in Patients with Early Rheumatoid Arthritis Receiving Methotrexate as First-Line Therapy: A Swedish Observational Nationwide Study. RMD Open.

[27] Tuano K.S., Seth N., Chinen J. (2021). Secondary Immunodeficiencies. Ann. Allergy. Asthma. Immunol..

[28] Onda K., Honma T., Masuyama K. (2023). Methotrexate-Related Adverse Events and Impact of Concomitant Treatment with Folic Acid and Tumor Necrosis Factor-Alpha Inhibitors: An Assessment Using the FDA Adverse Event Reporting System. Front. Pharmacol..

[29] Ding J., Ning Y., Bai Y., Xu X., Sun X., Qi C. (2019). β-Glucan Induces Autophagy in Dendritic Cells and Influences T-Cell Differentiation. Med. Microbiol. Immunol..

[30] Ali M.F., Dasari H., Van Keulen V.P., Carmona E.M. (2017). Canonical Stimulation of the NLRP3 Inflammasome by Fungal Antigens Links Innate and Adaptive B-Lymphocyte Responses by Modulating IL-1β and IgM Production. Front. Immunol..

[31] Zhu Z., He L., Bai Y., Xia L., Sun X., Qi C. (2023). Yeast β-Glucan Modulates Macrophages and Improves Antitumor NK-Cell Responses in Cancer. Clin. Exp. Immunol..

[32] He L., Bai Y., Xia L., Pan J., Sun X., Zhu Z., Ding J., Qi C., Tang C. (2022). Oral Administration of a Whole Glucan Particle (WGP)-Based Therapeutic Cancer Vaccine Targeting Macrophages Inhibits Tumor Growth. Cancer Immunol. Immunother..

[33] He Y., Du J., Dong Z. (2020). Myeloid Deletion of Phosphoinositide-Dependent Kinase-1 Enhances NK Cell-Mediated Antitumor Immunity by Mediating Macrophage Polarization. Oncoimmunology.

[34] Eisinger S., Sarhan D., Boura V.F., Ibarlucea-Benitez I., Tyystjärvi S., Oliynyk G., Arsenian-Henriksson M., Lane D., Wikström S.L., Kiessling R. (2020). Targeting a Scavenger Receptor on Tumor-Associated Macrophages Activates Tumor Cell Killing by Natural Killer Cells. Proc. Natl. Acad. Sci. USA.

[35] Khatua S., Simal-Gandara J., Acharya K. (2022). Understanding Immune-Modulatory Efficacy in Vitro. Chem. Biol. Interact..

[36] Megha K.B., Joseph X., Akhil V., Mohanan P.V. (2021). Cascade of Immune Mechanism and Consequences of Inflammatory Disorders. Phytomedicine.

[37] Noh H.-J., Park J.M., Kwon Y.J., Kim K., Park S.Y., Kim I., Lim J.H., Kim B.K., Kim B.-Y. (2022). Immunostimulatory Effect of Heat-Killed Probiotics on RAW264.7 Macrophages. J. Microbiol. Biotechnol..

[38] Liu Y., Yang J., Guo Z., Li Q., Zhang L., Zhao L., Zhou X. (2024). Immunomodulatory Effect of Cordyceps Militaris Polysaccharide on RAW 264.7 Macrophages by Regulating MAPK Signaling Pathways. Molecules.

[39] du Sert N.P., Hurst V., Ahluwalia A., Alam S., Avey M.T., Baker M., Browne W.J., Clark A., Cuthill I.C., Dirnagl U. (2020). The ARRIVE Guidelines 2.0: Updated Guidelines for Reporting Animal Research. PLoS Biol..

[40] Nair A., Jacob S. (2016). A Simple Practice Guide for Dose Conversion between Animals and Human. J. Basic Clin. Pharm..

