# Supplementary material for: Effects of Dietary Yeast β-1,3/1,6-D-Glucan on Immunomodulation in RAW 264.7 Cells and Methotrexate-Treated Rat Models
Source: Int J Mol Sci. 2024 Oct 14;25(20):11020. doi: 10.3390/ijms252011020 (PMC11508109; doi:10.3390/ijms252011020)
Supplement: Supplementary file 1 [file ijms-25-11020-s001.zip › ijms-3248229-supplementary.pdf]

Certificate of analysis

Certificate-no.	157404
Material	Yestimun® Beta-Glucan
Material-no.	100363
Batch number	22031296
Splitt-no.	0
Manufacturing date	09/06/2022
Best before	08/06/2027

**Parameter**	**Analytical Method**	**Specification**	**Analysis**
Dry matter	*ASU §64 LFGB L 02.09-4 / DIN 10453 (mod.)*<br>*Drying oven*	>= 94 %	96 %
Protein	*ASU § 64 LFGB L 01.00 10-1*<br>*Kjeldahl*	<= 4 %	2 %
Ash	*ASU §64 LFGB L01.00-77 (mod.)*<br>*Ash residue*	<= 2 %	1 %
(1,3)-(1,6)-ß-D-Glucan	*Megazyme, Enzymatic Yeast Beta-Glucan*	80...90 %	86 %
Gluten	*R-Biopharm; RIDASCREEN Gliadin; Art. Nr. R7001*<br>*ELISA*	<= 20 ppm	conform
Bulk density	*DIN EN ISO 60*<br>*Volumetry*	450...550 g/l	479 g/l
Arsenic	*ASU § 64 LFGB L 00.00-135 bzw. VDLUFA III 17.9.1*<br>*ICP-MS*	<= 0.20 mg/kg	conform
Lead	*ASU § 64 LFGB L 00.00-135 bzw. VDLUFA III 17.9.1*<br>*ICP-MS*	<= 0.20 mg/kg	conform
Cadmium	*ASU § 64 LFGB L 00.00-135 bzw. VDLUFA III 17.9.1*<br>*ICP-MS*	<= 0.100 mg/kg	conform
Mercury	*ASU § 64 LFGB L 00.00-135 bzw. VDLUFA III 17.4.3*<br>*ICP-MS*	<= 0.050 mg/kg	conform
Aerobic Plate Count		<= 1000 cfu / g	< 100 cfu / g
Yeasts		<= 25 cfu / g	< 10 cfu / g
Moulds		<= 25 cfu / g	< 10 cfu / g
Enterobacteriaceae		<= 100 cfu / g	< 10 cfu / g
Bacillus cereus		<= 100 cfu / g	< 10 cfu / g
Coliforms /g		negative	conform
E. coli /g		negative	conform
Staph. aureus / 25 g		negative	conform
Salmonellae / 25 g		negative	conform

Quality and sanitary controls were in agreement to specifications.

Bramsche, 06/09/22

Mr. M. Hebeke

Head of Quality Assurance

This certificate has been created electronically and is valid without signature.
Statement of the above data does not release the buyer from the legal obligation to examine the material on receipt.
These data cannot be construed as a legal warranty or guarantee of certain properties of the product for a specific application.
